# Fluorescent materials for pH sensing and imaging based on novel 1,4-diketopyrrolo-[3,4-*c*]pyrrole dyes†
†Electronic supplementary information (ESI) available: NMR and MS spectra, further sensor characteristics and sensor long-time performance. See DOI: 10.1039/c3tc31130aClick here for additional data file.



**DOI:** 10.1039/c3tc31130a

**Published:** 2013-08-05

**Authors:** Daniel Aigner, Birgit Ungerböck, Torsten Mayr, Robert Saf, Ingo Klimant, Sergey M. Borisov

**Affiliations:** a Institute of Analytical Chemistry and Food Chemistry , Graz University of Technology , Stremayrgasse 9 , Graz , Austria . Email: sergey.borisov@tugraz.at ; Tel: +43 316 873 32516; b Institute for Chemistry and Technology of Materials , Graz University of Technology , Stremayrgasse 9 , A-8010 Graz , Austria

## Abstract

New optical pH-sensors relying on 1,4-diketopyrrolo-[3,4-*c*]pyrroles (DPPs) as fluorescent pH-indicators are presented. Different polymer hydrogels are useful as immobilization matrices, achieving excellent sensitivity and good brightness in the resulting sensor. The operational pH can be tuned over a wide range (pH 5–12) by selecting the fine structure of the indicator and the matrix. A ratiometric sensor in the form of nanoparticles is also presented. It is suitable for RGB camera readout, and its practical applicability for fluorescence imaging in microfluidic systems is demonstrated. The indicators are synthesized starting from the commercially available DPP pigments by a straightforward concept employing chlorosulfonation and subsequent reaction with amines. Their sensitivity derives from two distinct mechanisms. At high pH (>9), they exhibit a remarkable alteration of both absorption and fluorescence spectra due to deprotonation of the lactam nitrogen atoms. If a phenolic group is introduced, highly effective fluorescence quenching at near-neutral pH occurs due to photoinduced electron transfer (PET) involving the phenolate form.

## Abstract 2

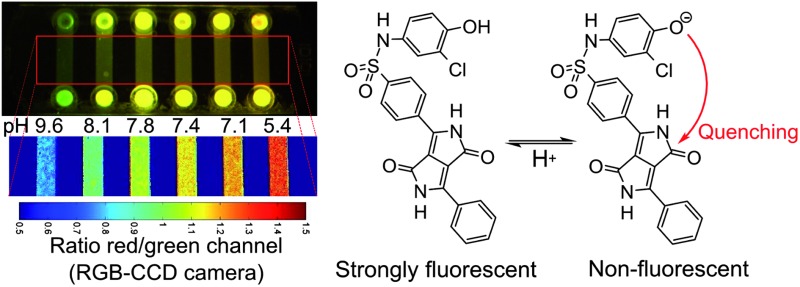
Fluorescent pH-sensors based on 1,4-diketopyrrolo-[3,4-*c*]pyrrole indicator dyes are presented. Their key advantages are excellent suitability for fluorescence imaging and tunability of the sensitive range.

## Introduction

pH is one of the key parameters in medical, environmental and life sciences. Despite the strong performance of electrochemical pH-sensors, optical pH-sensors have proved invaluable in numerous important applications. They possess numerous advantages including greater ease of miniaturization and the possibility of contactless measurement. Moreover, optical probes enable imaging applications.^1–5^ These features are particularly attractive in high-throughput screening and for probing small samples such as living cells or sub-cellular structures.^6,7^

Optical pH-sensors typically consist of a pH-sensitive dye (*i.e.* a pH-indicator) immobilized in a polymer matrix which has to provide suitable mechanical and adhesive properties, together with sufficient water uptake. Although most optical pH-indicators are essentially (de)protonatable chromophores,^8–10^ those with proton receptors separated from the chromophore have also found numerous applications. They are the most flexible in terms of rational dye design since the chromophore and the receptor can be selected independently. Most frequently, they take advantage of the photoinduced electron transfer (PET)^11–13^ process. Though PET is an extensively investigated effect, in most publications it is introduced by amine functionalities,^14–17^ while phenolic groups have attracted comparatively little attention. In 1997, Gareis *et al.* ^18^ presented a boron-dipyrromethene (BODIPY) pH-indicator with a phenolic proton receptor. Most comparable systems have relied on the BODIPY chromophore since then.^19–21^

Derivatives of 1,4-diketo-3,6-diphenylpyrrolo[3,4-*c*]pyrrole, often referred to as DPPs, are chemically stable, brightly fluorescent^22^ molecules that have found a variety of applications. While the parent compounds are commonly used pigments, the attachment of suitable substituents yields readily soluble fluorescent dyes. Alkylation of the lactam nitrogen atoms is most effective in this regard since hydrogen bonding interactions are suppressed. DPP-based dyes and pigments have been used as high-performance colorants in prints and inks, as components of solid-state dye lasers^23–27^ and more recently in the field of organic optoelectronics. Particularly, DPP-containing conjugated polymers^28–30^ and small molecules^31^ have found extensive use in Organic Field Effect Transistors (OFETs)^32,33^ and organic solar cells.^34,35^ DPP dyes are also particularly promising for the design of two-photon excitable fluorophores.^36,37^ A few DPP-based fluorescent probes and sensors for fluoride,^38^ cyanide,^39^ thiols^40^ and molecular hydrogen^41^ have been developed. The DPP-based probe presented by Yamagata *et al.* ^42^ is suitable for detecting strong acids in organic solvents, rather than measuring near-neutral pH in aqueous solution. Recently, we presented carbon dioxide sensors that exploit the deprotonation of the lactam nitrogen atoms in DPPs.^43^ The same mechanism is useful for the determination of comparatively high pH (>9), as will be demonstrated in this work. Furthermore, we present – to the best of our knowledge for the first time – DPP-based pH-sensors that operate at near-neutral pH. They rely on PET from phenolate groups to the chromophore.

## Results and discussion

**Fig. 1:**
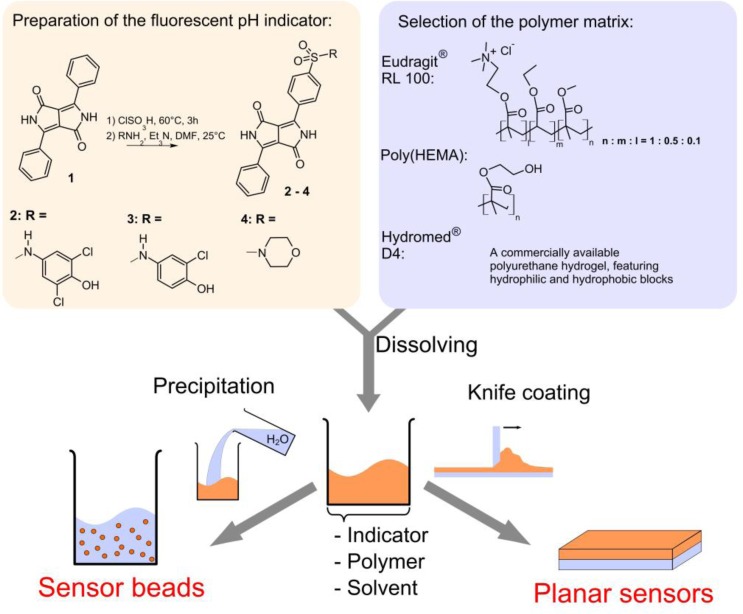
Scheme for the preparation of the fluorescence pH-sensors in this work.

The preparation of the new pH-indicators and sensors is shown in Fig. 1. The indicators **2** and **3** feature phenolic PET groups. **4** carries a morpholino group for solubilization and is an example of a DPP pH-indicator relying on deprotonation of the lactam nitrogen.

### Indicator syntheses

The indicators **2–4** can be easily prepared in a single step starting from commercially available 1,4-diketo-3,6-diphenylpyrrolo[3,4-*c*]pyrrole. Notably, the reaction conditions applied afforded only monosulfonated products. Doubly sulfonated products are formed under harsher reaction conditions.^43^ The intermediate, a sulfonyl chloride, can yield a large variety of sulfonamides, depending on the amine it is reacted with. The synthetic concept employed is simple and versatile as it is applicable for any class of chromophores that can withstand chlorosulfonation. It is useful for tagging a large variety of structures and is not limited to the preparation of pH-indicators, which has been the main focus of this work.

The modest yields (13–22%) are due to difficulties in purifying the products by column chromatography. They strongly bind to the stationary phase and are hard to elute completely. Nevertheless, all products could be easily isolated in sufficient amounts starting from the cheap commercial pigment.

### Indicator properties

Spectral properties of the DPP sulfonamides **2–4** in comparison with the starting material are listed in Table 1. The attachment of a sulfonamide group results in bathochromically shifted and less structured absorption bands, while the Stokes shifts are significantly enlarged. Despite that **2–4** are not *N*-substituted, their solubilities exceed 2 g l^–1^ in *N*,*N*-dimethylformamide and tetrahydrofuran. This is dramatically higher than for pigment **1** which is virtually insoluble in these solvents at 25 °C. Note that for the majority of applications requiring soluble DPPs, *N*-substituted derivatives are used. However, our preliminary experiments indicated that chlorosulfonation of a *N*,*N*′-dialkylated DPP (*N*,*N*′-di(2-ethylhexyl)-1,4-diketo-3,6-diphenylpyrrolo[3,4-*c*]pyrrole) and subsequent reaction with 4-amino-2,6-dichlorophenol did not yield a pH-indicator.

**Table 1:** Spectral properties of the DPP dyes in tetrahydrofuran: *λ*_max_ abs – wavelengths of the absorption maxima; *ε* – molar absorption coefficients; *λ*_max_ em – wavelengths of the fluorescence emission maxima; *Φ*_F_ – relative fluorescence quantum yield; n.d. – not determined; n.m. – not measureable (**2** and **3** are virtually non-fluorescent in the phenolate form). Acidic/basic denotes 0.1% (v/v) trifluoroacetic acid/1 mM tetrabutylammonium hydroxide

Dye	*λ*_max_ abs(*ε* × 10^–4^)/nm (M^–1^ cm^–1^), acidic/basic	*λ*_max_ em/nm, acidic/basic	*Φ*_F_, acidic/basic
**1**	468(2.95), 502(3.91)	514, 552	n.d.
**2**	509(2.23), 543(2.40)/575(1.88), 606(2.16)^*a*^	580/n.m.	0.70/n.m.
**3**	508(1.74), 541(1.86)/575(1.60), 606(1.86)^*a*^	577/n.m.	0.66/n.m.
**4**	509(2.02), 541(2.14)/391(0.95), 584(1.89), 619(2.39)	576/679	0.71/0.08

^*a*^The bathochromically shifted spectra correspond to the dianionic form (both phenol and lactam are deprotonated). The absorption of the monoanionic form (only phenol is deprotonated) is not shifted with respect to the acidic form (Fig. 3).

**Fig. 2:**
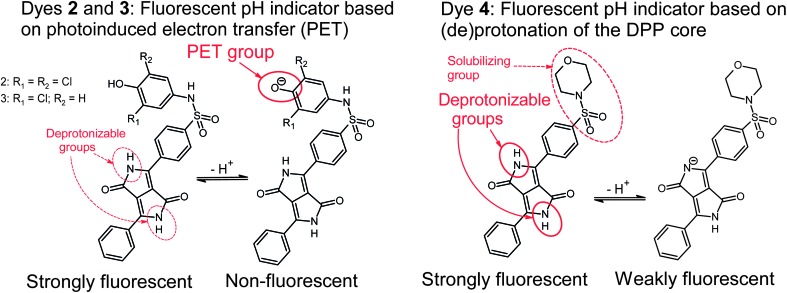
Mechanisms causing pH-sensitivity in the DPP-based indicators.

**Fig. 3:**
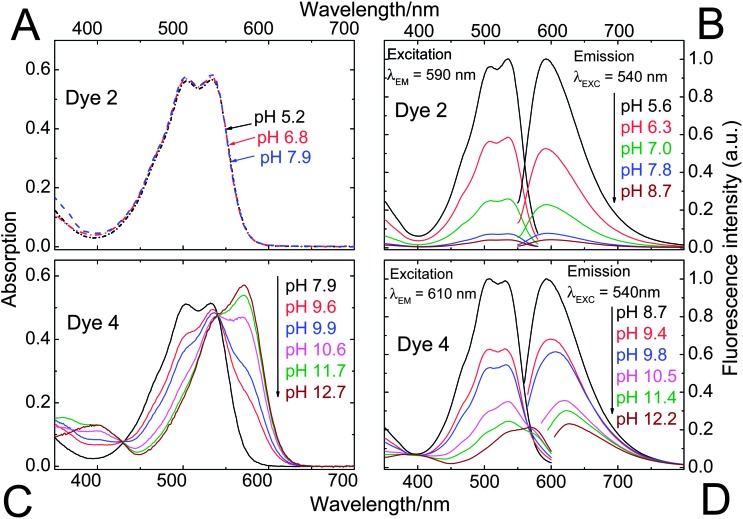
pH-dependent absorption (A and C) and fluorescence (B and D) spectra of the DPPs **2** and **4**. Since **2** and **3** feature virtually identical spectral properties and differ only by their sensitive pH ranges, **3** has been included in the electronic supplementary information (ESI; Fig. S1†) only. Spectra were recorded in ethanol–aqueous buffer (ionic strength 100 mM) solution 1 : 1 (v/v). pH values are those of the aqueous buffer used. DPP concentration was 20 μM for absorption and 4 μM for fluorescence measurements.

The pH-sensitivity of the DPP indicators is associated with two distinct mechanisms, as illustrated in Fig. 2. **2** and **3** are subject to photoinduced electron transfer (PET) when the phenolic group is deprotonated. The result is fluorescence quenching around the p*K*_a_ of the phenolic group (that is, pH 5.9–9.3, Table 2). No alteration of the absorption spectra at all is observed (Fig. 3), indicating that the effect is solely PET. Importantly, the quenching is extremely efficient (virtually no fluorescence from the deprotonated form is detected) which indicates that phenolates are suitable proton receptor groups for designing fluorescent pH-indicators. Thus they represent a very promising alternative to the much more common amino-based receptors.

**Fig. 4:**
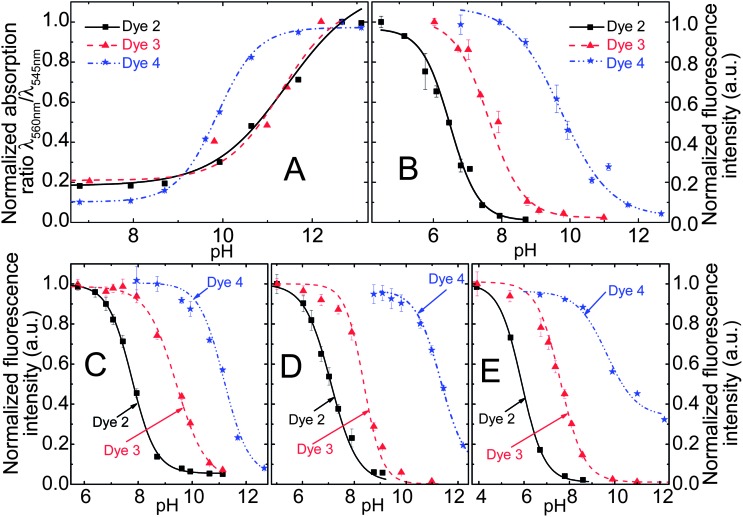
(A) pH calibration curves of the DPP indicators in ethanol–aqueous buffer (ionic strength 100 mM) solution 1 : 1 (v/v); pH values are those of the aqueous buffer used. (B) Corresponding fluorescence calibration curves for the solution (*λ*_EXC_ = 540 nm, observed at 595 nm); (C and D) pH calibration curves based on fluorescence in planar sensors (dye content 0.25%); (C) in D4® hydrogel; (D) in poly(2-hydroxyethylmethacrylate), (E) calibration curves in RL100 sensor beads dispersed in aqueous buffer (dye content 0.5% (w/w), bead concentration 0.2 mg ml^–1^).

**Table 2:** pH_1/2_, the pH values at which half of the pH-dependent signal change is effective, based on absorption (Abs.) and fluorescence (Fluo.), corresponding to the calibration curves in Fig. 4. n.d. denotes not determined

Dye	EtOH–H_2_O 1 : 1 (v/v)		D4		poly(HEMA)	RL100
	pH_1/2_ (Fluo.)	pH_1/2_ (Abs.)	pH_1/2_ (Fluo.)	pH_1/2_ (Abs.)	pH_1/2_ (Fluo.)	pH_1/2_ (Fluo.)
**2**	6.49^*a*^	11.3^*b*^	7.76^*a*^	n.d.^*c*^	7.08^*a*^	5.88^*a*^
**3**	7.63^*a*^	11.3^*b*^	9.34^*a*^	n.d.^*c*^	8.36^*a*^	7.62^*a*^
**4**	9.75^*b*^	9.88^*b*^	11.1^*b*^	11.6^*b*^	11.1^*b*^	9.65^*b*^

^*a*^Corresponds to deprotonation of the phenolic PET group.

^*b*^Corresponds to deprotonation of the lactam nitrogen.

^*c*^The doubly charged basic form is quickly leached out of the sensor.

Fluorescence quenching of **4** occurs at higher pH (9.7–11.6, Fig. 4) and is clearly accompanied by a bathochromic shift in absorption and fluorescence spectra. This effect is caused by deprotonation of the lactam nitrogen within the chromophore.^43^ Similar changes in the absorption spectra can also be observed for **2** and **3** at higher pH. The absorption spectra shown in Fig. 3C correspond to the neutral and the monoanionic form of **4**. Note that the monoanion exhibits weaker but clearly detectable fluorescence. Under more basic conditions, a further bathochromic shift is observable, which originates from partial deprotonation of the second lactam nitrogen, resulting in the dianion.

Notably, the sulfonamide moiety itself can also undergo deprotonation. Typical p*K*_a_ values would be in the range of 8–11 for structures comparable to **2** and **3**.^44,45^ Such a deprotonation mechanism may contribute to their pH-sensitivity to some extent. The anion formed, a sulfonimide, is in protolytic equilibrium with the phenolate form shown in Fig. 2. Note that the sulfonamide group in **4** cannot be deprotonated and its pH-sensitivity is thus related exclusively to lactam deprotonation.

**Fig. 5:**
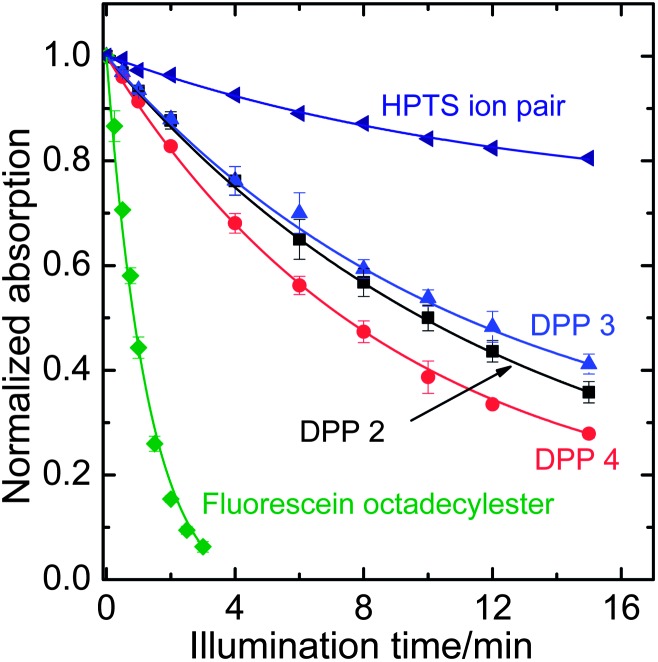
Photodegradation profiles of the DPP dyes, compared to fluorescein octadecyl ester and 1-hydroxypyrene-3,6,8-trisulfonate (HPTS) in the form of an ion pair with tetraoctylammonium. The solutions in tetrahydrofuran (for HPTS, 0.02 mM tetraoctylammonium hydroxide in the form of a 1 M methanolic solution was added) were illuminated with a 458 nm high-power LED array. The decay rates compared to **2** are 0.92 for **3**, 1.12 for **4**, 0.31 for the HPTS ion pair and 6.1 for FODE.

The photostability of the DPPs has been investigated and compared to reference dyes which represent two of the most commonly used types of pH-indicators. It is vastly superior to that of fluorescein octadecyl ester (Fig. 5). Compared to HPTS (8-hydroxypyrene-1,3,6-trisulfonate), a highly photostable pH-indicator, DPPs are degraded 3–4 times faster under the same illumination conditions. Photostability is greatly increased (dye degraded >20 times slower) in deoxygenated samples which implies an oxidative photo-degradation pathway or may involve photosensitized singlet oxygen. No degradation at all was observed for the phenolate form of **2**. This could be because PET causes fast quenching, leaving little time for degradation in the excited state.

### pH-sensing materials

The pH-indicators have been immobilized in polymer matrices to obtain pH-sensors. The host materials are Hydromed® D4 (a commercially available polyurethane-based hydrogel) and poly(2-hydroxyethylmethacrylate) (p(HEMA)) in the form of planar sensors and Eudragit® RL100 (a positively charged acrylate polymer) in the form of sensor nanobeads (typical average size 30 nm). The sensors are promising not only due to their high brightness and sensitivity, but in particular because their sensitive range can be tuned by selecting the indicator structure and the matrix (Table 2, Fig. 4). The PET-based indicators **2** and **3** can tackle pH 5–10. Monochlorinated **3** covers higher pH than dichlorinated **2**. The basic form of the indicator is negatively charged and thus most effectively stabilized by the cationic RL100 matrix and least effectively stabilized by the comparatively hydrophobic D4; this results in pH_1/2_ values increasing in the same order. The pH-range covered by **2** and **3** in those matrices meets the pH-range of interest for the most relevant applications of pH-sensors, namely medical applications (physiological pH 7–7.5), marine science (optimal pH 7.5–8.5) and biotechnological process monitoring (pH 5–7, depending on the process). **4** enables measurement at pH 9–12, a range that is more rarely addressed but is of importance to applications such as concrete quality testing. In this pH-range, the choice of already available pH-indicators is rather limited.

In practical applications, not only the sensitive range of a fluorescence sensor is of importance but also referencing possibilities. Referencing can make the signal independent of the optical path length, the efficiency of the light source and the guidance of light to the detector. Therefore, a ratiometric pH-sensor employing the commercially available coumarin Macrolex Yellow (3-(5-chloro-2-benzoxazolyl)-7-(diethylamino)-2*H*-1-benzopyran-2-one) as a reference dye has been developed. In this approach, the reference dye is excited, its excitation energy is partially transferred to the DPP pH-indicator dye by Förster resonance energy transfer (FRET) and the emission ratio between both dyes is detected. The referenced system is particularly promising since it can be read out with a simple RGB-CCD camera (Fig. S2 in the ESI†). Imaging with RGB cameras is becoming increasingly popular.^46–48^ Its sensitive range is very similar to that of the non-referenced analog, as demonstrated in Fig. S3.†

#### Planar sensors

**Fig. 6:**
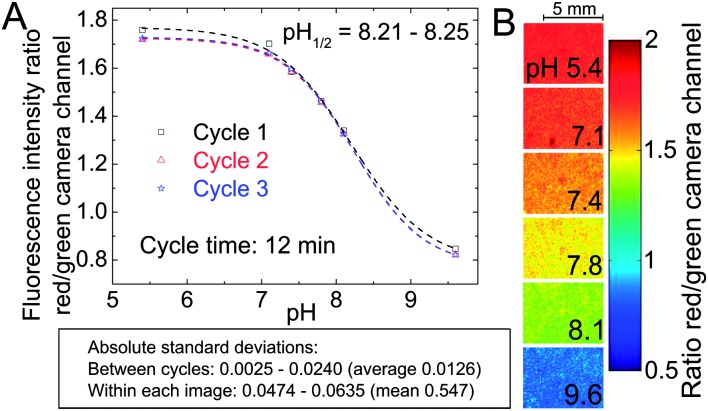
Fluorescence imaging with a planar ratiometric pH-sensor (7 μm thick) containing Macrolex Yellow (1.5% (w/w)) and pH-indicator **2** (0.5% (w/w)), read out with a RGB-CCD camera. The sensor was placed in a home-made flow-through cell and buffer solutions (ionic strength 100 mM) were applied (flow rate 1 ml min^–1^, 2 min for each buffer). A blue (458 nm) LED array in combination with a Schott BG 12 bandpass filter (350–465 nm) was used for excitation. Excitation light was excluded from the camera employing a Schott OG 515 nm long-pass filter. (A) Calibration curve, based on the ratio between red and green color channels; (B) corresponding false color images.

The usefulness of the planar ratiometric sensors is demonstrated in Fig. 6 – fluorescence imaging was successfully performed over an application time of >0.5 h. However, limitations were revealed when the long-term performance of the planar sensors was examined in a fiber-optic measurement setup with a LED light source. A significant decrease of the indicator signal is observable if a sensor based on **2** or **4** is measured over several hours (6% per hour for **2**, 14% per hour for **4**; visualized in the ESI, Fig. S4 and S5,† respectively). This signal drift is also present in the ratiometric system. It is strongly intensified when measurements are carried out under continuous illumination and it is accompanied by a diminishment in the DPP absorption band. This implies that photo-degradation is an important factor for the signal drift. However, no significant dependence on illumination time was found if that time is generally short (a fraction of <1% of the overall measurement time) and minimal emission intensity of the light source is applied. That suggests that other effects like dye aggregation or migration can also cause unwanted signal drift and become dominant at lower light intensities. Consequently, the DPP-based pH-sensors in the planar format are limited to applications requiring short times and low light densities.

#### Sensor beads

**Fig. 7:**
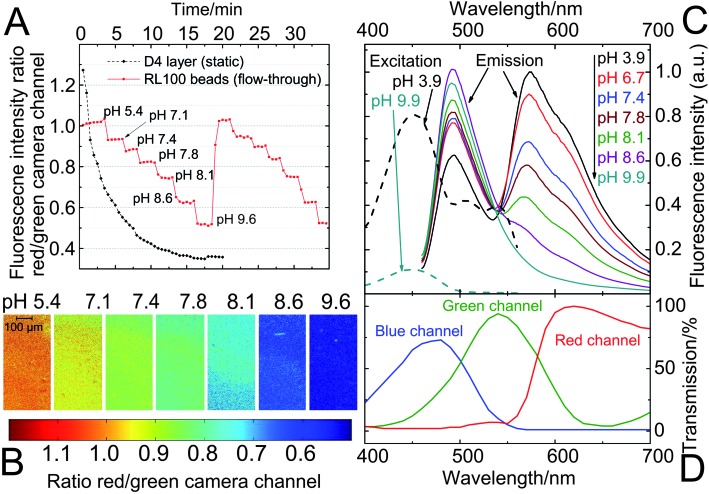
pH-imaging in a microfluidic system using a fluorescence microscope, employing ratiometric pH-nanosensor beads containing indicator **3** (1% w/w) and Macrolex® yellow (1.25% w/w) in RL100 polymer. The sensors were read out employing a RGB-CCD camera; (A) response curve of the bead suspensions; each measurement point was recorded after illumination for 2 s (the corresponding calibration curve can be found in Fig. S6 in the ESI†). They are compared to the performance of a planar sensor (static, placed on a microscope slide; composition as specified in Fig. 6) under the same measurement conditions; (B) images of the sensor bead suspensions corresponding to measurement A (cycle 1) – the ratio between red and green channels is visualized; (C) pH-dependent fluorescence spectra (emission excited at 450 nm; excitation observed at 570 nm) of the sensor beads; (D) spectral characteristics of the RGB-CCD camera.

**Fig. 8:**
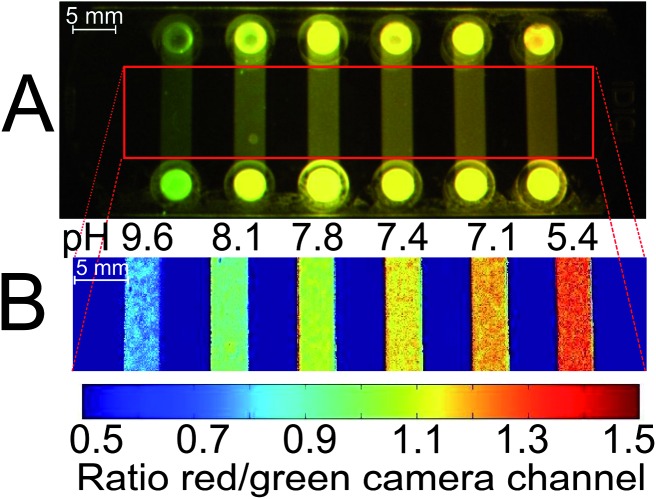
Example of a possible application of the DPP-based ratiometric pH-nanosensor beads in high-throughput measurements, using pH-imaging in a microfluidic system. pH can be determined simultaneously in every chip compartment. The pH-sensor beads (as specified in Fig. 7) in aqueous buffer of defined pH were read out employing a RGB-CCD camera. Excitation source and filters were the same as in Fig. 6. (A) Photographic image; (B) false color image of the chip. At the channel inlets, local higher optical pathlengths cause deviations in fluorescence measurements due to overexposure. When red and green color channels exhibit maximum intensities, the ratio of these channels is 1. For the sake of clarity, the inlets are not shown in (B).

In contrast to planar sensors which are static, sensor beads can be dispersed in the sample. Therefore, in all fluidic applications the beads are continuously renewed. When the residence time of each individual nanosensor is short, signal drifts within the sensor do not become noticeable even if the actual application time is long. As a result, photodegradation can be negligible in the case of sensor beads, while the same sensing materials used as planar sensors suffer from a signal drift. An example for this is given in Fig. 7A where the performances of DPP-based ratiometric pH-sensors in both formats are compared using a fluorescence microscope. Owing to the high light density typical for fluorescence microscopy, the signal of the planar sensor is dramatically affected by photo-bleaching and measurement is rendered essentially impossible. On the other hand, the performance of the RL100 sensor beads in a fluidic system remains unperturbed over the whole measurement time. Consequently, while the DPP-based planar sensors and sensor beads can both be useful for macroscopic fluorescence imaging (Fig. 6), the use of beads is imperative in fluorescence microscopy. Fig. 7 also demonstrates the applicability of the sensor beads in a microfluidic system. Note that imaging in microfluidic systems enables high-throughput measurements, for instance in medical applications. RGB cameras are generally simple readout systems available at low cost and were demonstrated to be suitable for pH-imaging in microfluidics.^49^ To underline the suitability of the DPP-based sensor beads in this regard, they were used in a microfluidic system that allows parallelization (Fig. 8).

## Conclusions

In conclusion, new pH-sensors have been presented that exclusively rely on fluorescent dyes and host polymers which are either commercially available or can be prepared from commercially available compounds in a single, simple reaction step. By appropriate selection of the sensor components, one can tune the sensitive pH-range of the material to cover a broad region (pH 5–12). This pH-region meets the requirements of a vast majority of pH-sensor applications. While sensors in the planar format are limited to short-time applications, sensitive nanobeads in Eudragit® RL100 polymer are most promising for practical applications in (micro)fluidic systems and even for fluorescence imaging and microscopy. By taking advantage of a ratiometric approach, good compatibility with a simple RGB camera for readout is accomplished.

Beyond the presented new pH-sensors, this work highlighted the usefulness of the tools employed for rational dye design. The phenolate proton receptors have been demonstrated to be very efficient quenchers operating *via* photoinduced electron transfer and represent a promising alternative to the more commonly used amino-receptors. They can be used in combination with other fluorophores in order to match the requirements of a particular application considering spectral properties or indicator stability. The receptors have been attached by a simple concept, *i.e.* chlorosulfonation and subsequent reaction with amines. Following this concept, a variety of functionalities can be tagged to DPPs, making them suitable for probing analytes other than pH or targeting particular biomolecular structures.

## Experimental

### Materials and methods

1,4-Diketo-3,6-diphenylpyrrolo[3,4-*c*]pyrrole (Irgazin Scarlet) was purchased from Kremer Pigmente (; http://kremer-pigmente.de/en), and Macrolex Fluorescent Yellow from Simon and Werner GmbH (; http://www.simon-und-werner.de). 4-Amino-2-chlorophenol was from TCI Europe (; http://www.tcichemicals.com), and 4-amino-2,6-dichlorophenol from ABCR (; http://www.abcr.de). Solvents used for work-up and purification (synthesis grade) as well as buffer salts were supplied by Carl Roth (; http://www.roth.de). Deuterated solvents were obtained from Eurisotop (; http://www.eurisotop.com), and silica gel from Acros (; http://www.fishersci.com). Polyurethane hydrogel D4® was from CardioTech (; http://www.cardiotech-inc.com), poly(2-hydroxyethylmethacrylate) (MW = 150 000 g mol^–1^) from Polysciences Inc. (; http://www.polysciences.com), and Eudragit® RL100 from Evonik Industries (; http://corporate.evonik.de). All other chemicals were from Sigma-Aldrich (; http://www.sigmaaldrich.com). The poly(ethylene glycol terephthalate) support (Mylar®) was from Goodfellow (; http://www.goodfellow.com). Fluorescein octadecyl ester was prepared according to the literature procedure.^50^

NMR spectra were recorded on a 300 MHz instrument (Bruker) with TMS as a standard. MALDI-TOF mass spectra were taken on a Micromass TofSpec 2E in reflectron mode at an accelerating voltage of +20 kV. Absorption measurements were performed on a Cary 50 UV-Vis spectrophotometer from Varian (http://www.varianinc.com). Fluorescence spectra were recorded on a Hitachi F-7000 spectrofluorimeter (; http://www.hitachi.com). Relative fluorescence quantum yields were determined at 25 °C using rhodamine 101 (*Φ*_F_ = 0.98 in ethanol^51^) as a standard. Photostability measurements were performed by irradiating the samples with the light of a 458 nm high-power LED array (10 W input power, ; http://www.led-tech.de) focused through a lens purchased from Edmund Optics. The photodegradation profiles were obtained by monitoring the absorption spectra.

pH-imaging was performed using a RGB-CCD camera (Marlin F201C, Allied Vision Technologies, http://www.stemmer-imaging.de) equipped with a Xenoplan 1.4/23 objective lens (; http://www.schneiderkreuznach.com). For images taken on the fluorescence microscope (Zeiss Axiovert 25 CFL, ; http://corporate.zeiss.com), a blue ultrabright LED with emission maximum at *λ* = 450 nm (Luxeon lambert emitter, blue, 5 W) was applied as the excitation light source and combined with a filter set-up consisting of Linos DT blue/Linos DC blue/Schott OG 515 (LINOS Photonics, Göttingen, Germany; Schott, ; http://www.schott.com) as the excitation filter/dichromatic mirror/barrier filter, respectively. Image acquisition was performed with the software AVT SmartView (; http://www.alliedvisiontec.com). Matlab R2008a (; http://www.mathworks.com) was used for image processing. The color channels of the obtained images were separated and the ratiometric images were obtained by dividing the red by the green channel.

Microfluidic flow-through experiments were performed using a custom made flow cell or a 6 channel μ-Slide (ibidi μ-Slide VI ^0.4^, ; http://ibidi.com), which was connected to a syringe pump (model 540060, TSE systems, ; http://www.tse-systems.com).

The pH of the buffer solutions was controlled by a digital pH-meter (InoLab pH/ion, WTW GmbH & Co. KG, http://www.wtw.com) calibrated at 25 °C with standard buffers of pH 7.0 and 4.0. The buffers were adjusted to a constant ionic strength of 100 mM using sodium chloride as the background electrolyte.

### Indicator synthesis

#### 1,4-Diketo-3-((4-[*N*-(3,5-dichloro-4-hydroxyphenyl)amino]sulfonyl)phenyl)-6-phenylpyrrolo[3,4-*c*]pyrrole (**2**)

1,4-Diketo-3,6-diphenylpyrrolo[3,4-*c*]pyrrole (500 mg, 1.73 mmol) was heated in chlorosulfuric acid (3 ml) to 60 °C. After 3 h, the mixture was allowed to cool to RT and was added dropwise onto ice cubes. The deep orange precipitate was filtered using a Büchner funnel, rinsed with cold H_2_O (0 °C) until pH was neutral and dried by applying a rotary vane pump for 0.5 h. The obtained sulfonyl chloride was dissolved in dry *N*,*N*-dimethylformamide (30 ml), and 4-amino-2,6-dichlorophenol (1.25 g, 7.02 mmol, 4 equiv.) and triethylamine (1.94 ml, 13.9 mmol) were added. The mixture was stirred for 2.5 h at RT, and then 1 M aqueous HCl (150 ml) was added. The precipitate was washed with water, dried and purified by column chromatography (silica gel, 40–63 μm) with ethyl acetate/chloroform 75/25 as an eluent. Yield: 188 mg (21%). Mp: decomposition at >260 °C. UV/vis absorption: *λ*_max_(tetrahydrofuran)/nm 246 (*ε*/dm^3^ mol^–1^ cm^–1^ 37 000), 291 (32 100), 509 (22 300) and 543 (24 000). IR absorption: *ν*_max_/cm^–1^ 3426 and 3320 (NH), 3222 (OH), 3030–3160 and 2800–2980 (CH), 1676, 1630 and 1595 (CO), 1555, 1488, 1395, 1331, 1283, 1219, 1156, 1088, 986, 893, 843, 811, 757, 726, 701, 645, 605, 552, 470. NMR (300 MHz, DMSO-*d*_6_, TMS): *δ*_H_ = 11.47 (1H, s, Ar-H), 10.44 (1H, s, ArOH), 10.11 (1H, s, SO_2_NH), 8.3–8.7 (2H, br s, CONH), 8.33 (3H, d, *J* = 8.4 Hz, Ar-H), 8.26 (1H, dd, *J*_1_ = 7.7 Hz, *J*_2_ = 1.1 Hz, Ar-H), 7.70–7.86 (4H, m, Ar-H), 7.09 (2H, s, Ar-H). *δ*_C_ = 175.67, 166.14 (C=O); 147.20, 146.29, 139.39, 139.02, 135.80, 133.54, 132.49, 131.34, 130.49, 130.16, 129.17, 127.65, 126.25, 124.73, 122.64, 120.99, 110.81, 100.35 (aromatic). MALDI-TOF: *m*/*z* [MH^+^] 528.0190 found, 528.0188 calcd.

#### 1,4-Diketo-3-((4-[*N*-(3-chloro-4-hydroxyphenyl)amino]sulfonyl)phenyl)-6-phenylpyrrolo[3,4-*c*]pyrrole (**3**)

**3** was prepared according to the same procedure as **2**, and 4-amino-2-chlorophenol (4 equiv.) was reacted with the sulfonyl chloride. Column chromatography was performed with ethyl acetate/ethanol/25% aqueous NH_3_ 93/7/0.33 as an eluent. Yield 116 mg (14%). Mp: decomposition at >270 °C. UV/vis absorption: *λ*_max_(tetrahydrofuran)/nm 245 (*ε*/dm^3^ mol^–1^ cm^–1^ 27 200), 291 (23 800), 508 (17 400) and 541 (18 600). *ν*_max_/cm^–1^ 3407 and 3314 (NH), 3225 (OH), 3030–3160 and 2800–2980 (CH), 2360, 2339, 1677, 1635 and 1593 (CO), 1557, 1507, 1487, 1405, 1332, 1287, 1202, 1156, 1088, 1053, 953, 895, 843, 820, 757, 726, 701, 614, 597, 544, 496. NMR (300 MHz, DMSO-*d*_6_, TMS): *δ*_H_ = 11.45 (1H, s, Ar-H), 10.10 (2H, d, ArOH, SO_2_NH), 8.3–8.7 (2H, br s, CONH), 8.31 (3H, dd, *J*_1_ = 8.1 Hz, *J*_2_ = 2.1 Hz, Ar-H), 8.26 (1H, dd, *J*_1_ = 7.7 Hz, *J*_2_ = 1.4 Hz, Ar-H), 7.70–7.86 (4H, m, Ar-H), 7.06 (1H, d, *J* = 2.3 Hz, Ar-H), 6.89 (1H, dd, *J*_1_ = 8.7 Hz, *J*_2_ = 2.4 Hz, Ar-H), 6.85 (1H, d, *J* = 8.5 Hz, Ar-H). *δ*_C_ = 175.64, 166.15 (C=O); 150.50, 147.15, 139.79, 139.16, 135.81, 133.26, 132.46, 131.31, 130.48, 129.24, 128.99, 127.65, 126.67, 124.71, 123.23, 121.78, 119.54, 116.90, 110.75, 100.36 (aromatic). MALDI-TOF: *m*/*z* [MH^+^] 494.0600 found, 494.0577 calcd.

#### 1,4-Diketo-3-((4-(1-morpholinyl)sulfonyl)phenyl)-6-phenylpyrrolo[3,4-*c*]pyrrole (**4**)

1,4-Diketo-3,6-diphenylpyrrolo[3,4-*c*]pyrrole (300 mg, 1.04 mmol) was heated in chlorosulfuric acid (3 ml) to 60 °C. After 3 h, the mixture was allowed to cool to RT and added dropwise onto morpholine (10 ml). The deep red mixture was stirred for 10 min and H_2_O (80 ml) was added. The precipitate formed was thoroughly washed with water, dried and purified by column chromatography (silica gel, 40–63 μm) with methanol/chloroform 5/95 as an eluent. Yield 170 mg (22%). Mp: >300 °C. UV/vis absorption: *λ*_max_(tetrahydrofuran)/nm 247 (*ε*/dm^3^ mol^–1^ cm^–1^ 28 000), 292 (27 900), 509 (20 000) and 541 (21 400). IR absorption: *ν*_max_/cm^–1^ 3419 and 3309 (NH), 3030–3170 and 2780–2980 (CH), 2368, 2339, 1665, 1627 and 1597 (CO), 1554, 1486, 1449, 1436, 1340, 1325, 1284, 1260, 1161, 1114, 1095, 938, 839, 747, 701, 668, 612, 595, 536, 469. NMR (300 MHz, DMSO-*d*_6_, TMS): *δ*_H_ = 11.58 (1H, s, Ar-H), 8.3–8.8 (2H, CONH), 8.50 (2H, d, *J* = 8.7 Hz, Ar-H), 8.36 (1H, d, *J* = 7.8 Hz, Ar-H), 8.30 (1H, dd, *J*_1_ = 7.8 Hz, *J*_2_ = 1.1 Hz, Ar-H), 7.85 (3H, dt, *J*_1_ = 8.4 Hz, *J*_2_ = 1.9 Hz, Ar-H), 7.77 (1H, t, *J* = 7.5 Hz, Ar-H), 3.67 (4H, t, *J* = 4.2 Hz, OCH_2_), 2.95 (4H, t, *J* = 4.1 Hz, ArNCH_2_). *δ*_C_ = 175.71, 166.19 (C=O); 147.27, 138.99, 135.81, 135.01, 133.76, 132.49, 131.34, 130.48, 129.15, 127.66, 127.26, 124.73, 110.90, 100.41 (aromatic); 65.29 (C–O); 45.92 (C–N). MALDI-TOF: *m*/*z* [MH^+^] 438.10 found, 438.11 calcd.

### Preparation of planar sensors

A “cocktail” containing the indicator dye (0.16 mg), hydrogel D4/p(HEMA) (41 mg) and ethanol–water 9 : 1 (v/v) (500 μl) was knife-coated on a dust-free Mylar support to obtain an ≈7 μm thick layer after solvent evaporation. Ratiometric sensors were prepared analogously, using 0.2 mg of **2**, 0.6 mg of Macrolex Yellow and tetrahydrofuran (461 μl) as a solvent.

### Preparation of sensor nanoparticles

Eudragit RL100 (100 mg) was dissolved in acetone (50 ml), and indicator dye (1 mg) and Macrolex Yellow (1.25 mg) were added. Water (300 ml) was added quickly (5 s). Acetone was removed on a rotary evaporator and the particle suspension was concentrated to a volume of 50 ml.
