# Supplementary material for: Fluorescent materials for pH sensing and imaging based on novel 1,4-diketopyrrolo-[3,4-c]pyrrole dyes
Source: J Mater Chem C Mater. 2013 Aug 5;1(36):5685–93. doi: 10.1039/c3tc31130a (PMC3778741; doi:10.1039/c3tc31130a)
Supplement: Supplementary file 1 [file TC-001-c3tc31130a-s001.pdf]

Electronic Supplementary Information

Fluorescent materials for pH sensing and imaging based on novel 1,4-diketopyrrolo-[3,4-c]pyrrole dyes

Daniel Aigner [a], Birgit Ungerböck [a], Torsten Mayr [a], Robert Saf [b], Ingo Klimant [a] and Sergey M Borisov[a*]

[a] Institute of Analytical Chemistry and Food Chemistry, Graz University of Technology, Stremayrgasse 9, A-8010 Graz, Austria

[b] Institute for Chemistry and Technology of Materials, Graz University of Technology, Stremayrgasse 9, A-8010 Graz, Austria

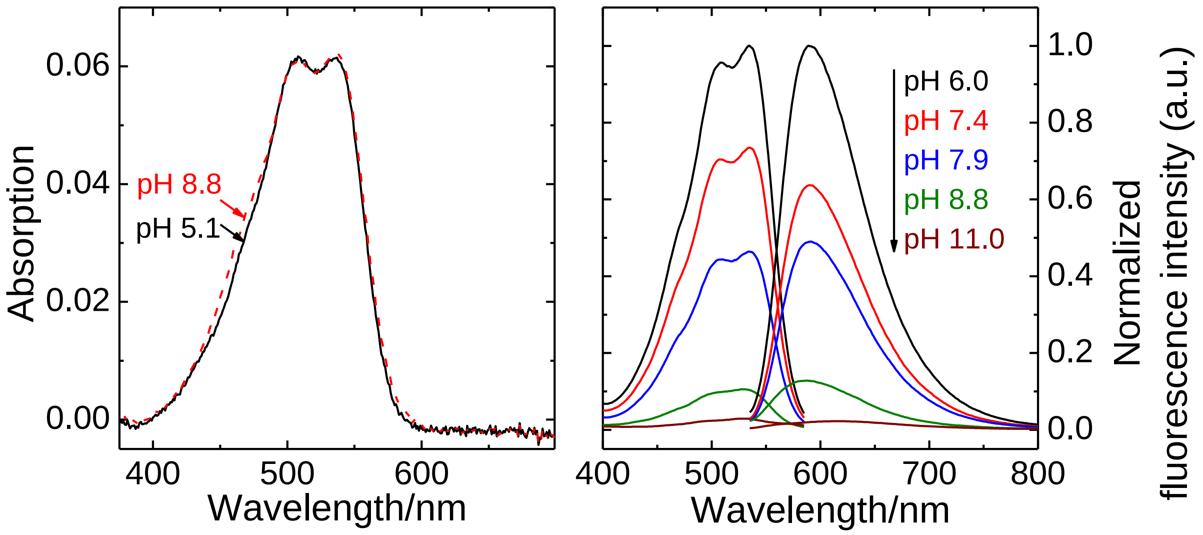

Figure S1: pH-dependent absorption and fluorescence spectra of **3**. Spectra were recorded in ethanol/aqueous buffer (ionic strength 100 mM) solution 1:1 (V/V). pH values are those of the aqueous buffer used. DPP concentration was 20 µM for absorption and 4 µM for fluorescence measurements.

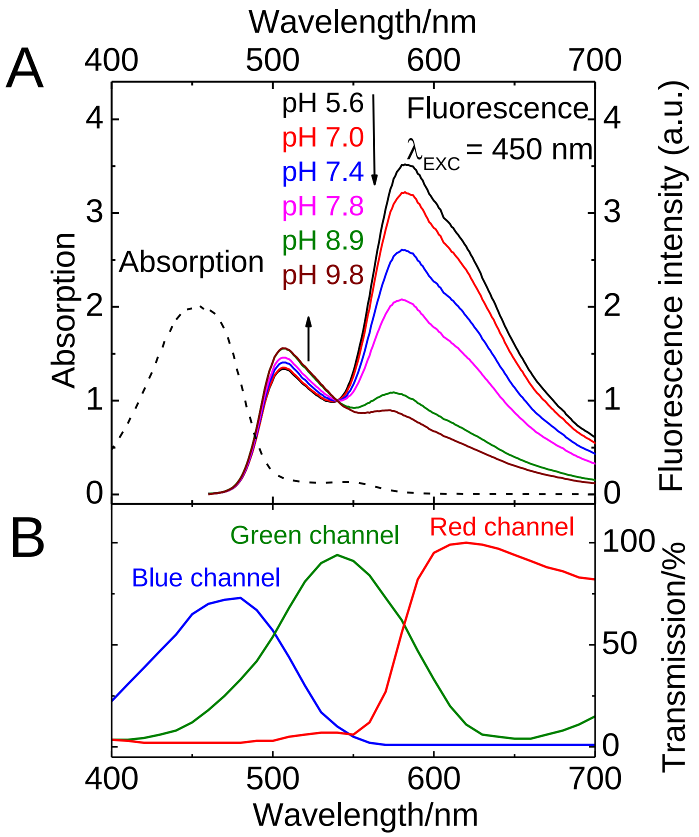

Figure S2: A: pH-dependent fluorescence spectra a planar ratiometric sensor containing Macrolex Yellow (1.5% (w/w)) and DPP pH-indicator **2** (0.5% (w/w)). **B**: Spectral characteristics of the RGB CCD camera.

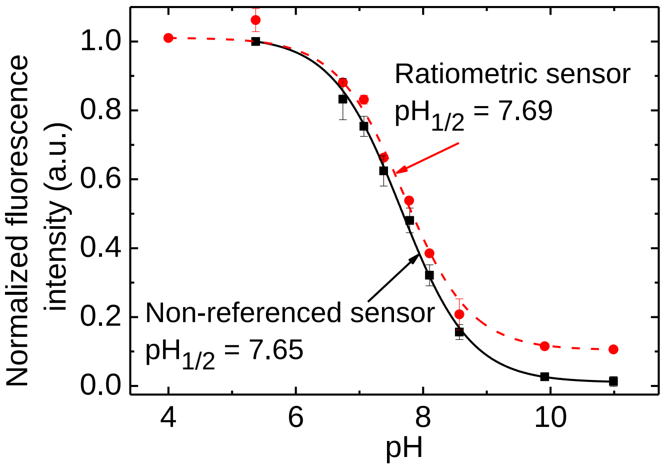

Figure S3: pH calibration curve of the ratiometric sensor beads, *i.e.* dye **3** (1% w/w) and Macrolex Yellow (reference dye; 1.25% w/w) in RL100 (bead content 2 mg / ml in aqueous buffer of ionic strength 100 mM), compared to beads containing only **3** (0.5% w/w) – this non-referenced system is also included into fig. 4 (main text).

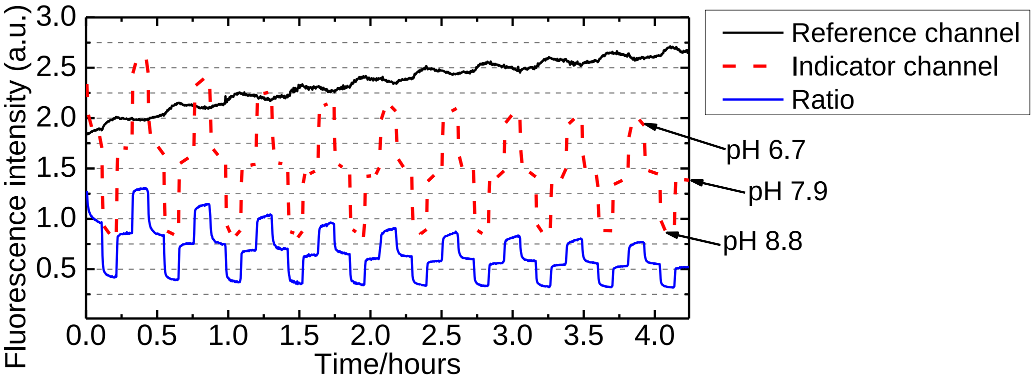

Figure S4: Long-time performance of a planar ratiometric sensor containing Indicator **2** (0.5% w/w) and Macrolex Yellow (1.5% w/w) in D4 hydrogel, excited with a 450 nm LED (Roithner, www.roithner-laser.com) combined with a Schott BG 12 bandpass filter (350 - 465 nm). The reference channel was equipped with 520 / 40 nm bandpass filter, the indicator channel with a 600 / 50 nm bandpass filter (both from Edmund optics, www.edmundoptics.de). Both channels were equipped with separate PMT detectors, *i.e.* do not represent absolute brightness ratios. The planar sensor was placed in a home-made flow-through cell and 100 mM buffer was passed through the cell, flow rate 1ml / min. Cell temperature was kept constant at 25 °C. The sensors were interrogated with a two-phase lock-in amplifier (SR830, Stanford Research Inc., www.thinksrs.com) equipped with a PMT detector (H5701-02, Hamamatsu, www.sales.hamamatsu.com). Illumination time was 1% of the measurement time.

This journal is © The Royal Society of Chemistry 2013

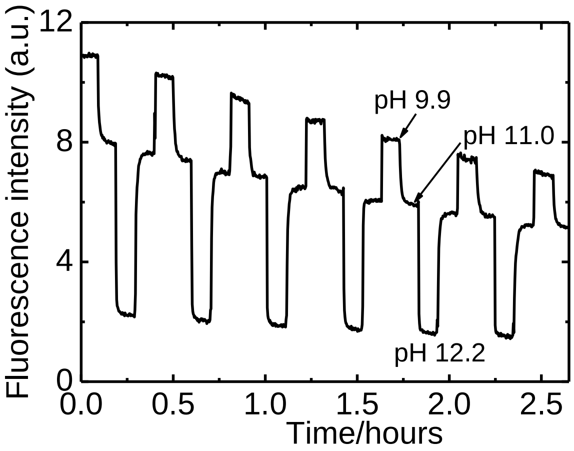

Figure S5: Long-time performance of a planar sensor containing **4** (0.4% w/w) in D4 hydrogel, excited with a 525 nm LED combined with a 520 / 40 nm bandpass filter (Edmund Optics) and a Schott OG 550 nm longpass filter before the detector. Measurement was carried out as stated near fig. S3.

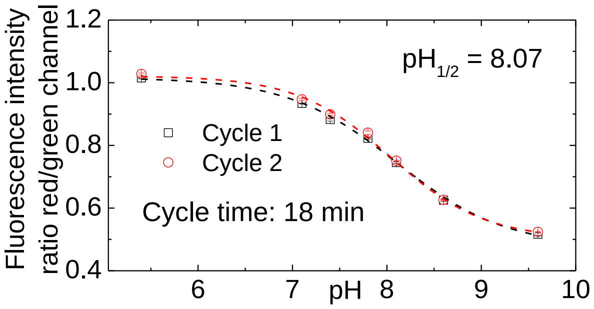

Standard deviations
Between cycles: 0.0027 - 0.0104 (average 0.0066)
Within each image (error bar): 0.0010 - 0.0056 (mean 0.0031)

Figure S6: pH-calibration curve of pH-nanosensor beads containing dye **3** (1% w/w) and Macrolex yellow (reference dye; 1.25% w/w) in RL100 polymer. The beads were read out under the fluorescence microscope employing a RGB-CCD camera. Response curve and images are shown in fig. 7.

NMR spectra

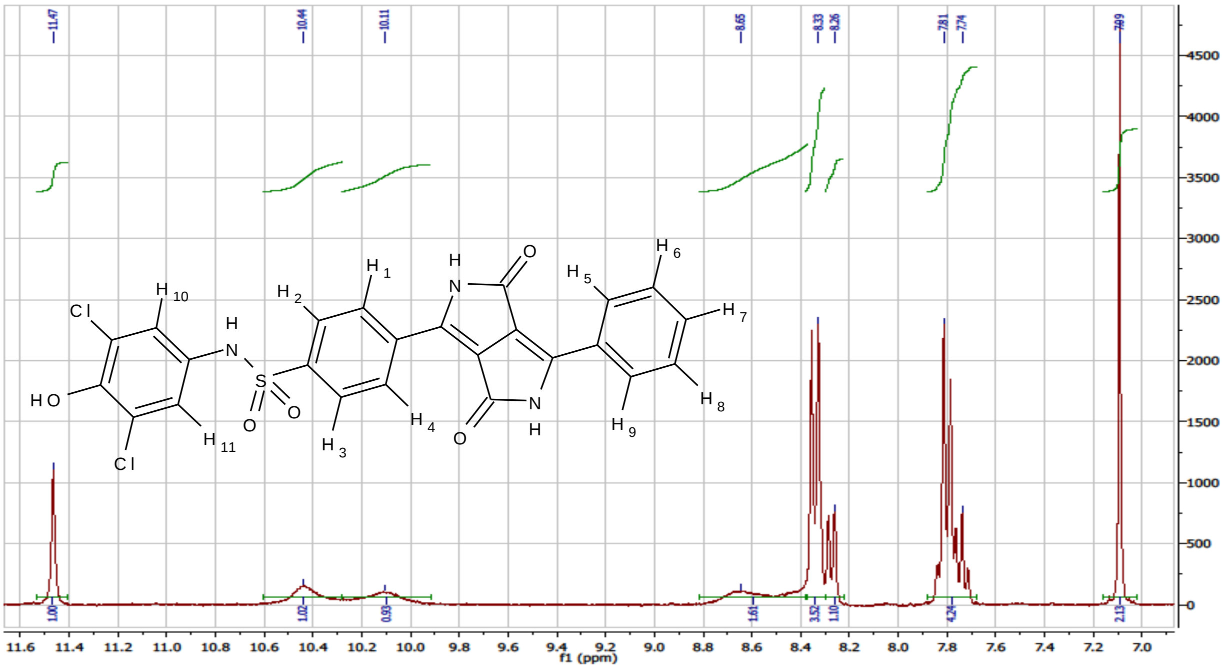

Figure S7: ^{1}H-NMR spectrum of **2** (300 MHz, DMSO-*d6*, TMS): δ_H = 11.47 (1 H, s, Ar-H(9)), 10.44 (1 H, s, ArOH), 10.11 (1 H, s, SO_2NH), 8.3 - 8.7 (2 H, CONH), 8.33 (3 H, d, J = 8.4 Hz, Ar-H(2,3,5)), 8.26 (1 H, dd, J_1 = 7.7 Hz, J_2 = 1.1 Hz, Ar-H(8)), 7.70 - 7.86 (4 H, m, Ar-H(1,4,6,7)), 7.09 (2 H, s, Ar-H(10,11)).

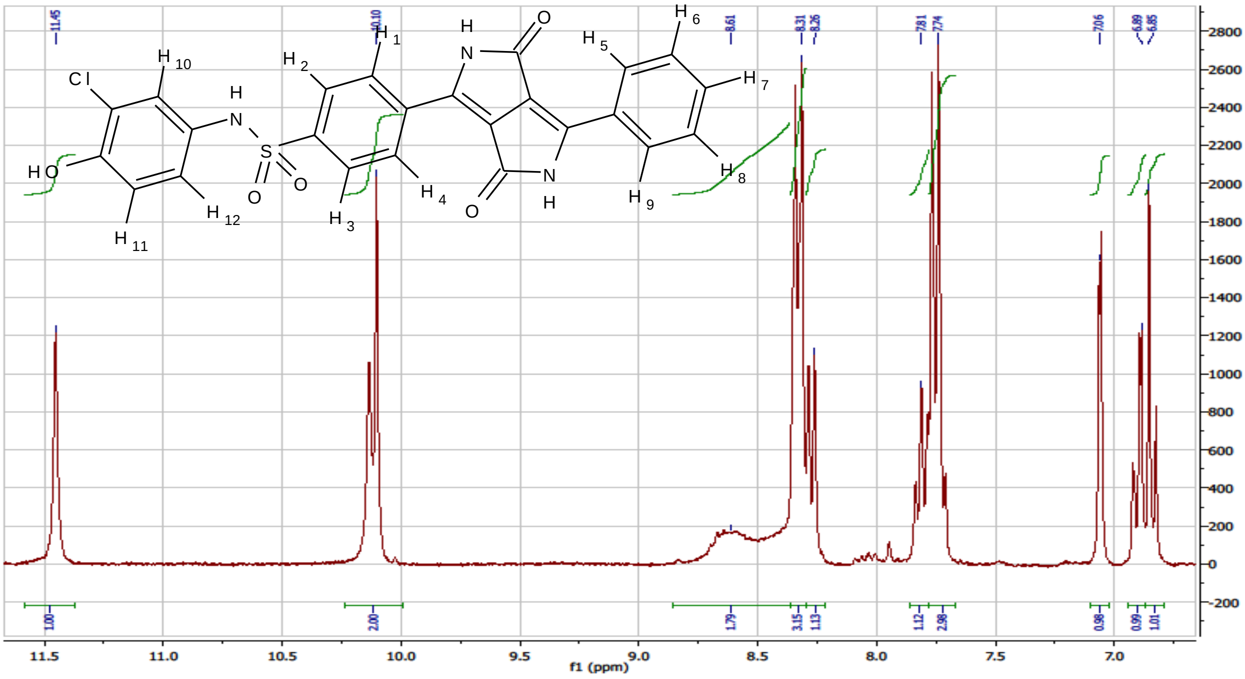

Figure S8: ^{1}H-NMR spectrum of **3** (300 MHz, DMSO-*d6*, TMS): δ_H = 11.45 (1 H, s, Ar-H(9)), 10.10 (2 H, d, ArOH, SO_2NH), 8.3 - 8.7 (2 H, CONH), 8.31 (3 H, dd, J_1 = 8.1 Hz, J_2 = 2.1 Hz, Ar-H(2,3,5)), 8.26 (1 H, dd, J_1 = 7.7 Hz, J_2 = 1.4 Hz, Ar-H(8)), 7.70 - 7.86 (4 H, m, Ar-H(1,4,6,7)), 7.06 (1 H, d, J = 2.3 Hz, Ar-H(10)), 6.89 (1 H, dd, J_1 = 8.7 Hz, J_2 = 2.4 Hz, Ar-H(12)), 6.85 (1 H, d, J = 8.5 Hz, Ar-H(11)).

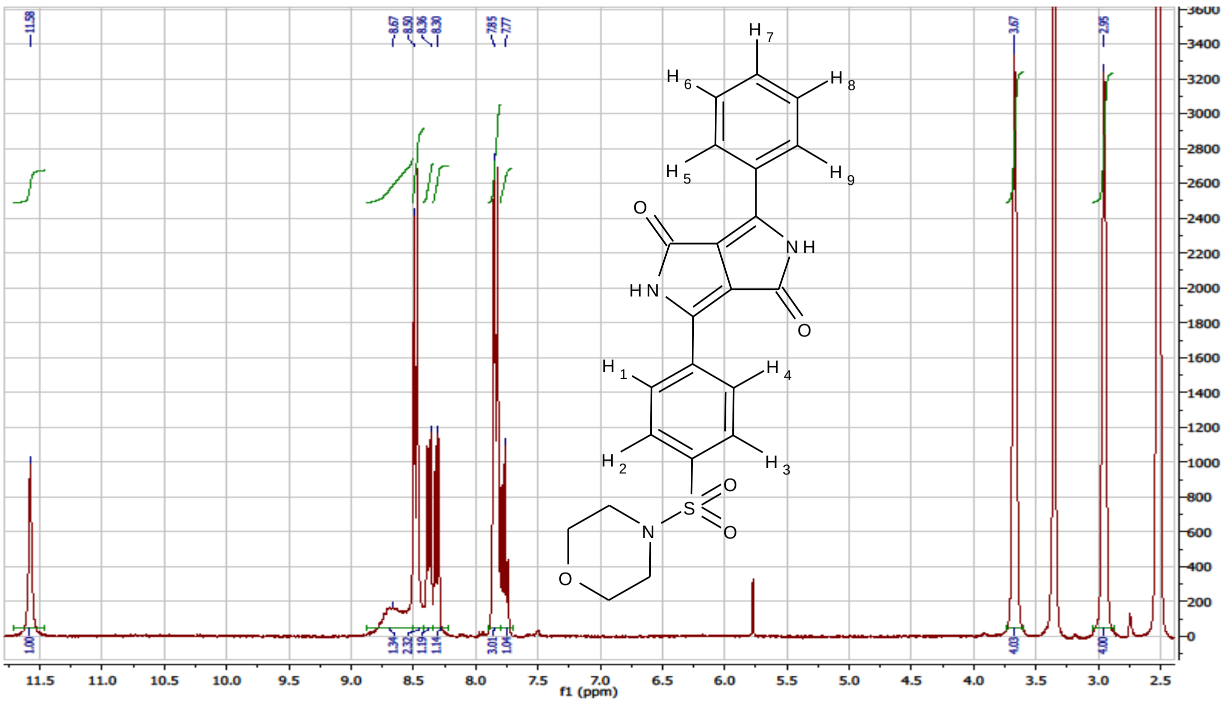

Figure S9: ^{1}H-NMR spectrum of **4** (300 MHz, DMSO-*d6*, TMS): δ_H = 11.58 (1 H, s, Ar-H(9)), 8.3 - 8.8 (2 H, CONH), 8.50 (2 H, d, J = 8.7 Hz, Ar-H(2,3)), 8.36 (1 H, d, J = 7.8 Hz, Ar-H(5)), 8.30 (1 H, dd, J_1 = 7.8 Hz, J_2 = 1.1 Hz, Ar-H(8)), 7.85 (3 H, dt, J_1 = 8.4 Hz, J_2 = 1.9 Hz, Ar-H(1,4,6)), 7.77 (1 H, t, J = 7.5 Hz, Ar-H(7)), 3.67 (4 H, t, J = 4.2 Hz, OCH_2), 2.95 (4 H, t, J = 4.1 Hz, $ArNCH_2$).

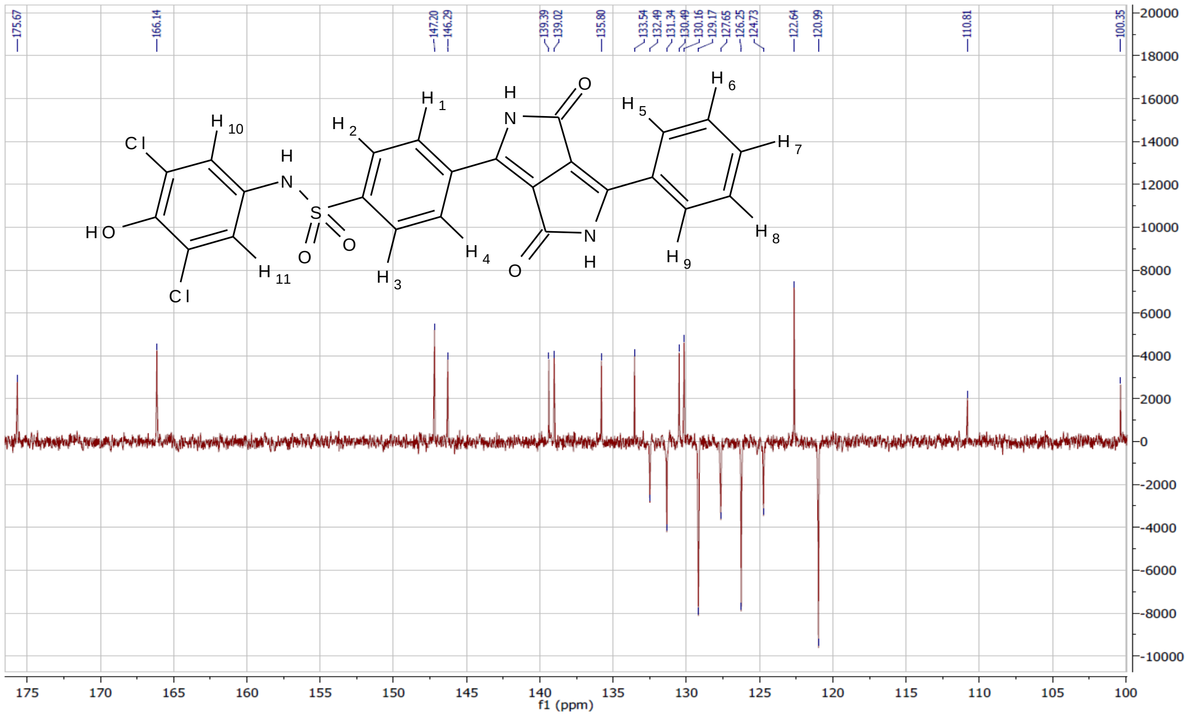

Figure S10: ^{13}C-APT-NMR spectrum of **2** (300 MHz, DMSO-*d6*, TMS): δ_C = 175.67, 166.14 (C=O); 147.20, 146.29, 139.39, 139.02, 135.80, 133.54 (C_{Ar}); 132.49 (C_{Ar}-H_6), 131.34 (C_{Ar}-H_7); 130.49, 130.16 (C_{Ar}); 129.17 (2C, C_{Ar}-H_2, C_{Ar}-H_3), 127.65 (C_{Ar}-H_8), 126.25 (2C, C_{Ar}-H_1, C_{Ar}-H_4), 124.73 (C_{Ar}-H_5); 122.64 (2C, C_{Ar}); 120.99 (2C, C_{Ar}-H_{10}, C_{Ar}-H_{11}); 110.81, 100.35 (C_{Ar}).

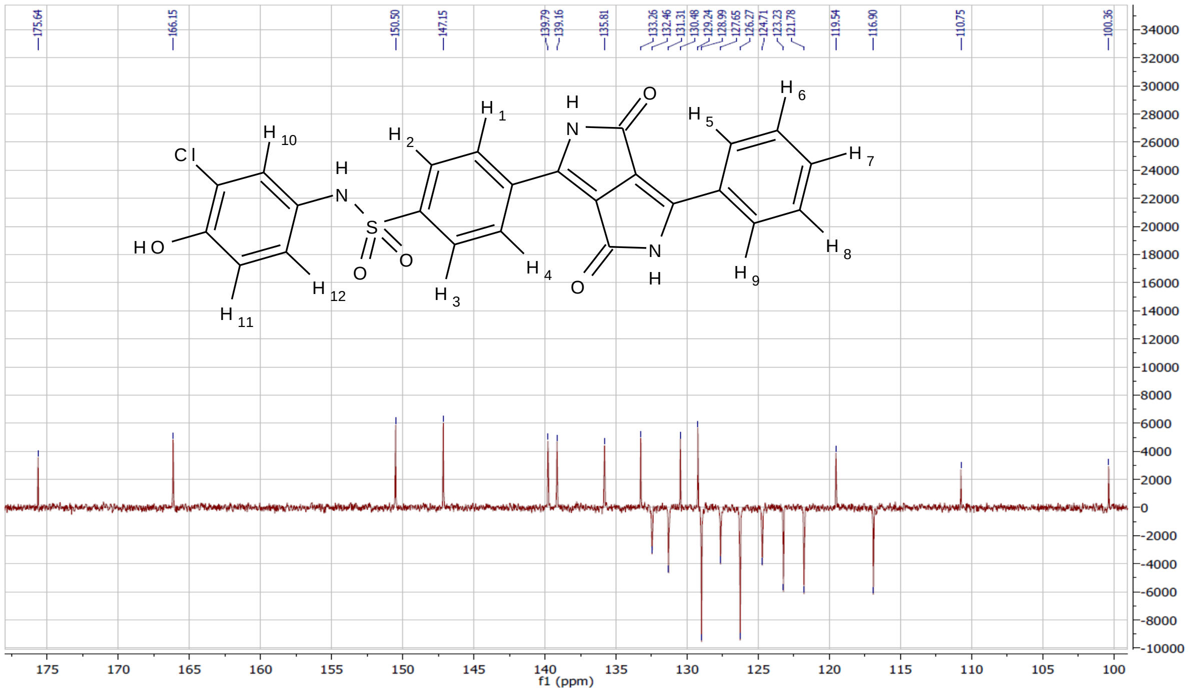

Figure S11: ^{13}C-APT-NMR spectrum of **3** (300 MHz, DMSO-*d6*, TMS): δ_C = 175.64, 166.15 (C=O); 150.50, 147.15, 139.79, 139.16, 135.81, 133.26 (C_{Ar}); 132.46 (C_{Ar}-H_6), 131.31 (C_{Ar}-H_7); 130.48, 129.24 (C_{Ar}); 128.99 (2C, C_{Ar}-H_2, C_{Ar}-H_3), 127.65 (C_{Ar}-H_8), 126.67 (2C, C_{Ar}-H_1, C_{Ar}-H_4), 124.71 (C_{Ar}-H_5), 123.23 (C_{Ar}-H_{10}), 121.78 (C_{Ar}-H_{12}); 119.54 (C_{Ar}); 116.90 (C_{Ar}-H_{11}); 110.75, 100.36 (C_{Ar}).

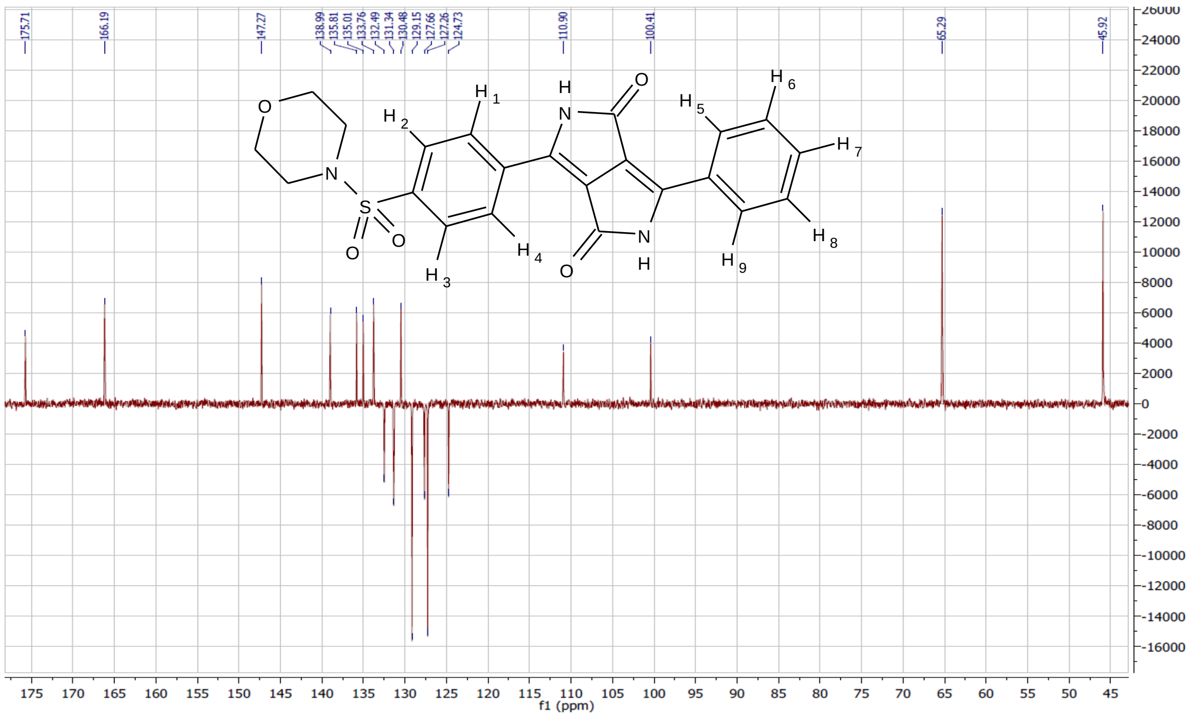

Figure S12: ^{13}C-APT-NMR spectrum of **4** (300 MHz, DMSO-*d6*, TMS): δ_C = 175.71, 166.19 (C=O); 147.27, 138.99, 135.81, 135.01, 133.76 (C_{Ar}); 132.49 (C_{Ar}-H_6), 131.34 (C_{Ar}-H_7); 130.48, (C_{Ar}); 129.15 (2C, C_{Ar}-H_2, C_{Ar}-H_3), 127.66 (C_{Ar}-H_8), 127.26 (2C, C_{Ar}-H_1, C_{Ar}-H_4), 124.73 (C_{Ar}-H_5); 110.90, 100.41 (C_{Ar}); 65.29 (C-O); 45.92 (C-N).

MALDI-TOF spectra:

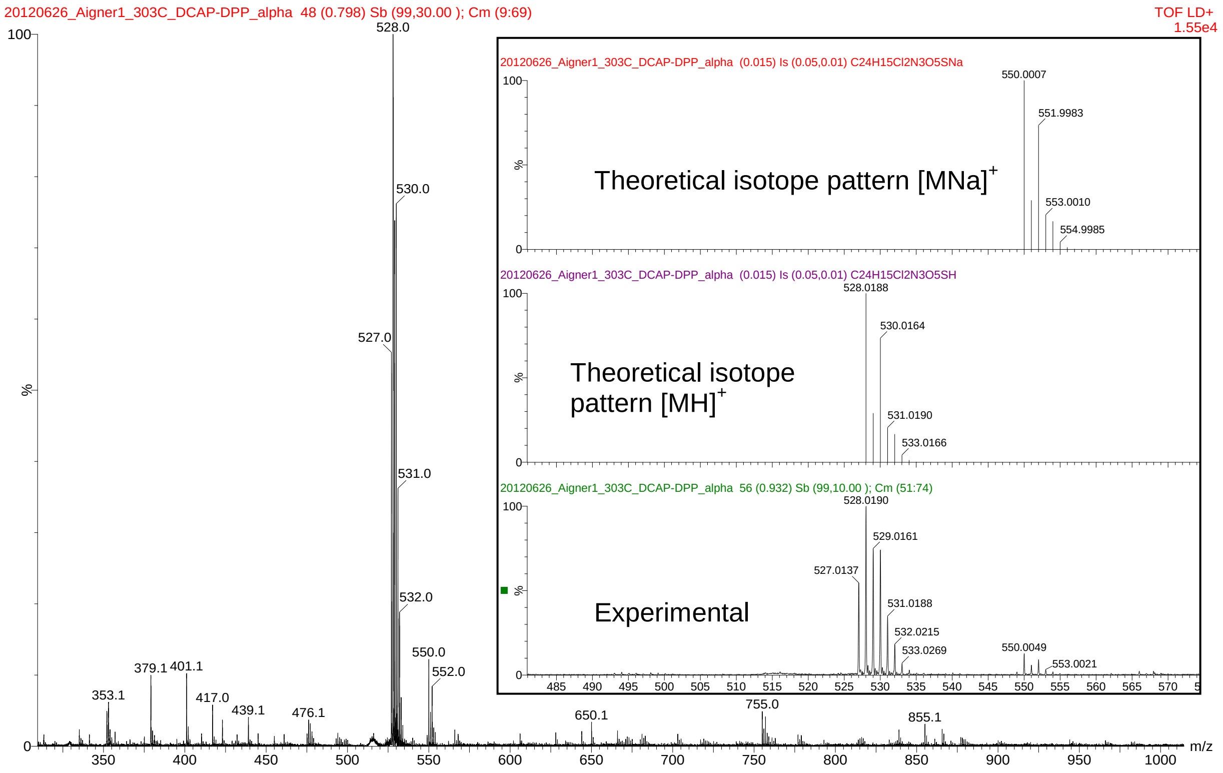

Figure S13: MALDI-TOF spectrum of **2**

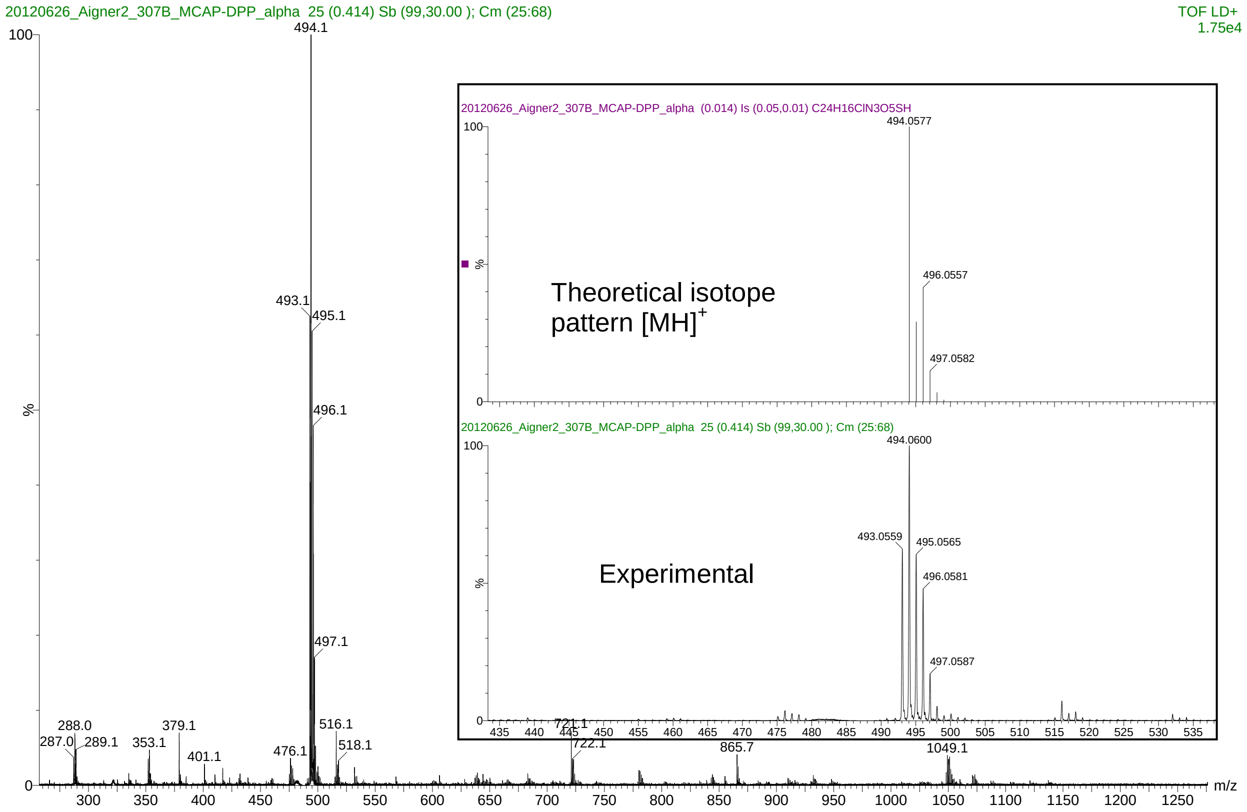

Figure S14: MALDI-TOF spectrum of **3**

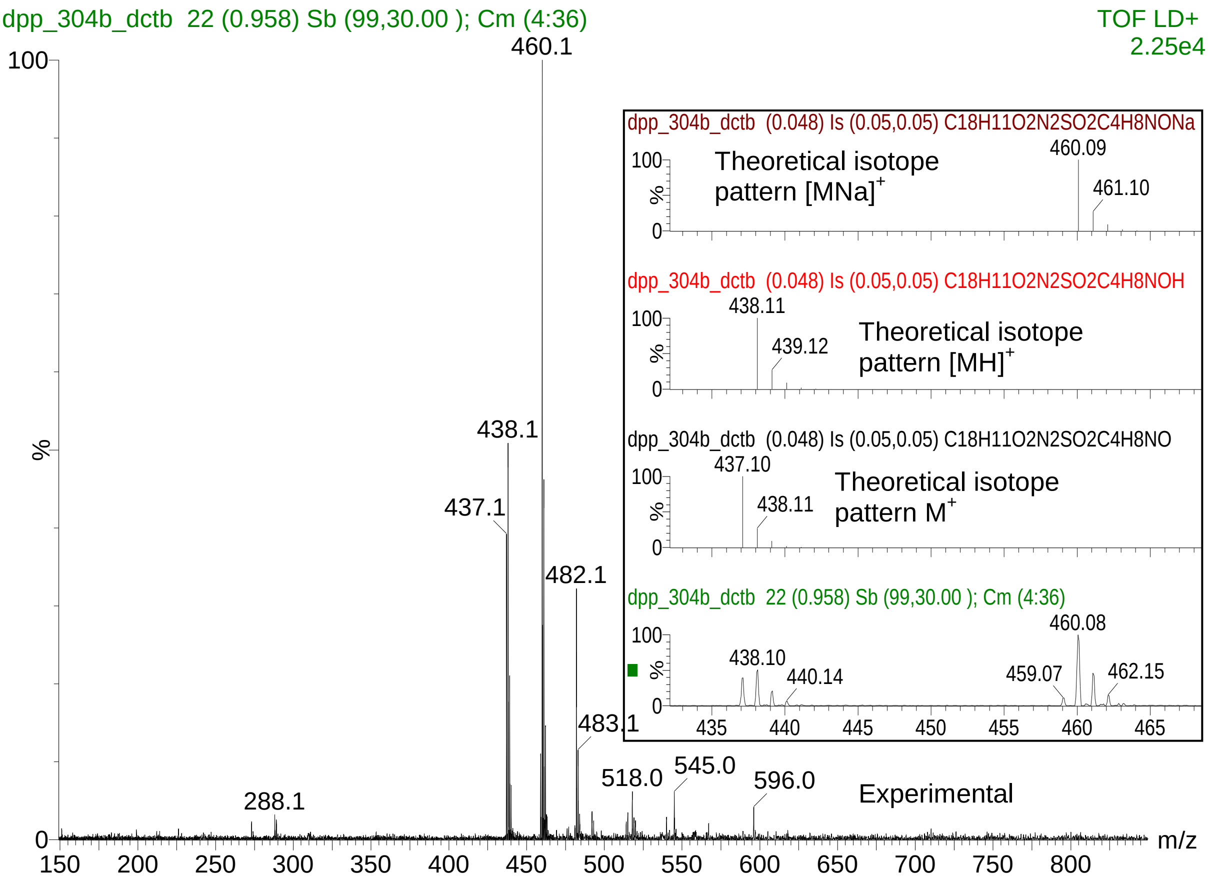

Figure S15: MALDI-TOF spectrum of **4**

Full UV/VIS absorption spectra (230 – 1000 nm):

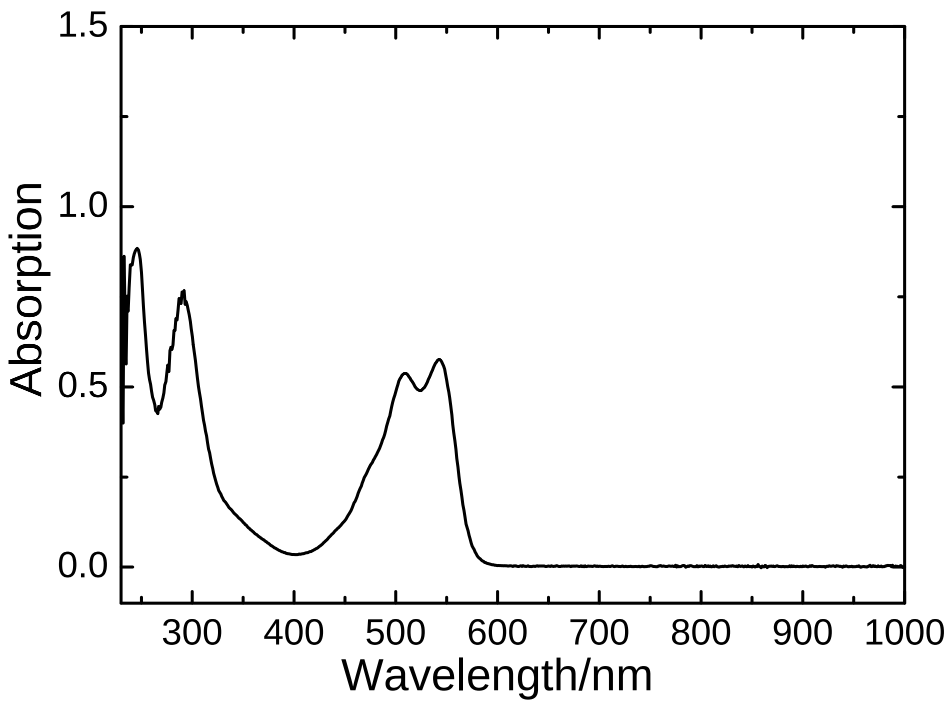

Figure S16: UV/VIS absorption spectrum of **2** in tetrahydrofuran

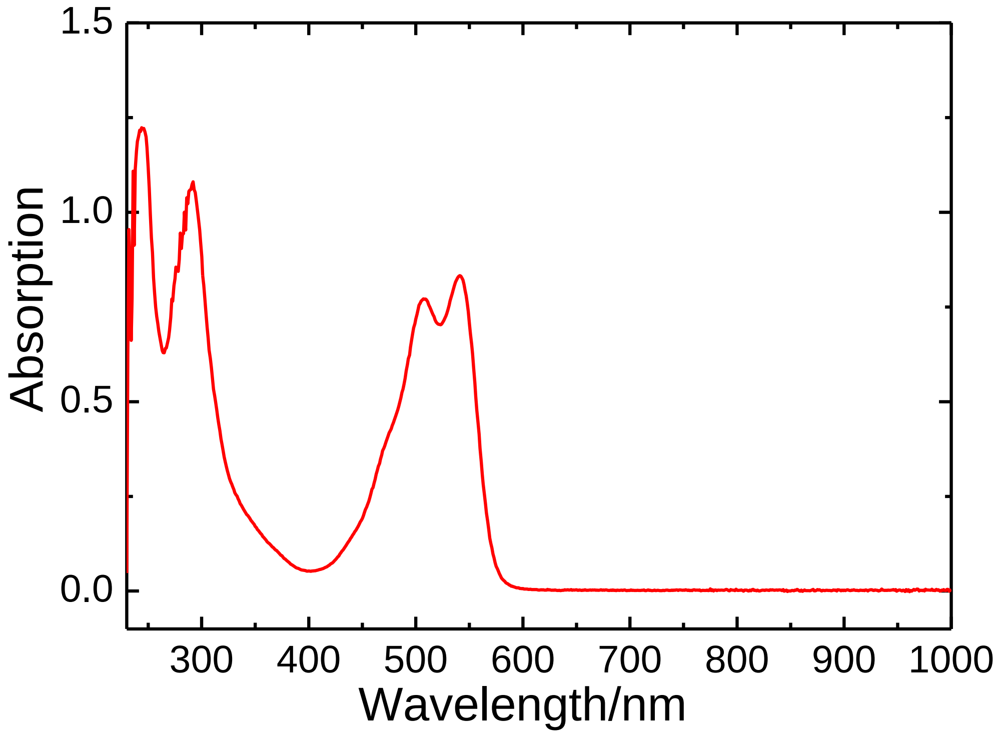

Figure S17: UV/VIS absorption spectrum of **3** in tetrahydrofuran

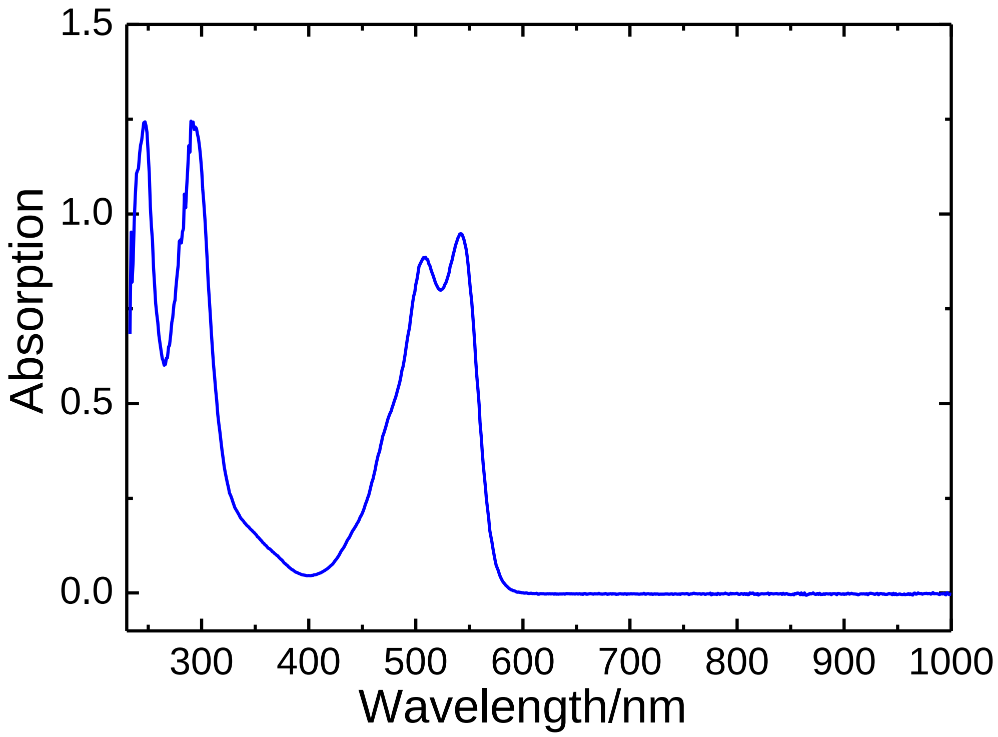

Figure S18: UV/VIS absorption spectrum of **4** in tetrahydrofuran

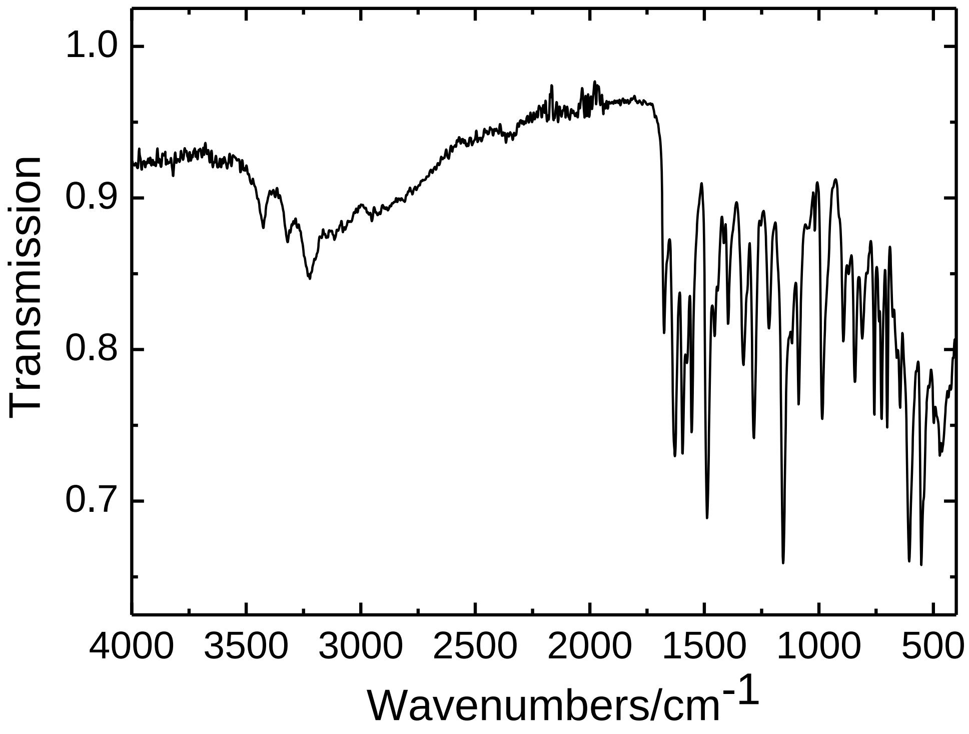

Figure S19: ATR-IR spectrum of **2**

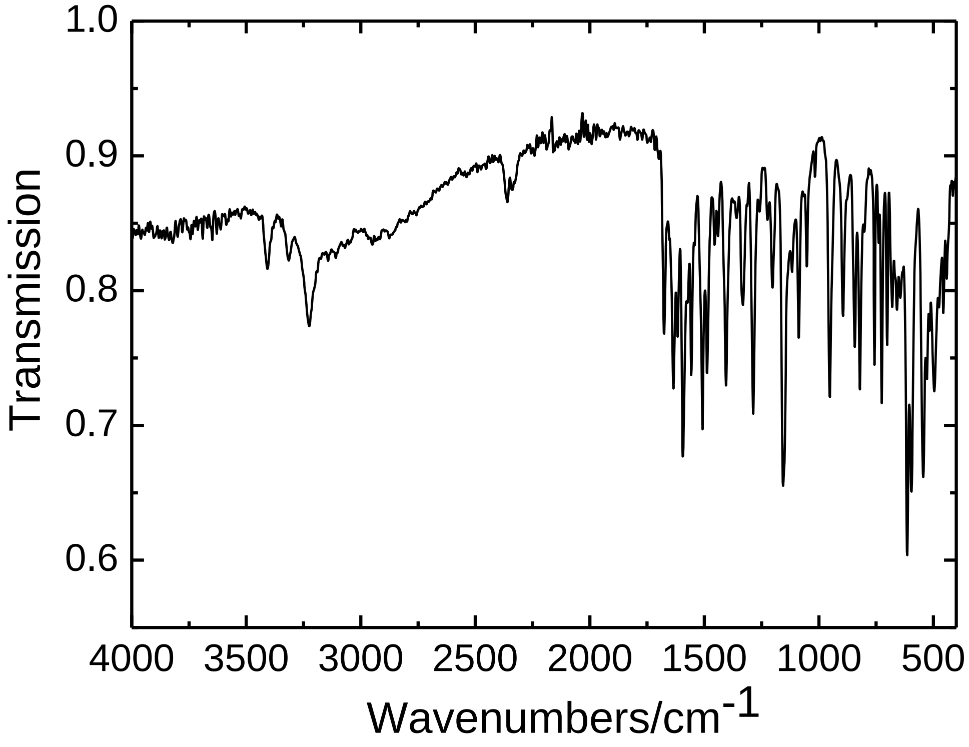

Figure S20: ATR-IR spectrum of **3**

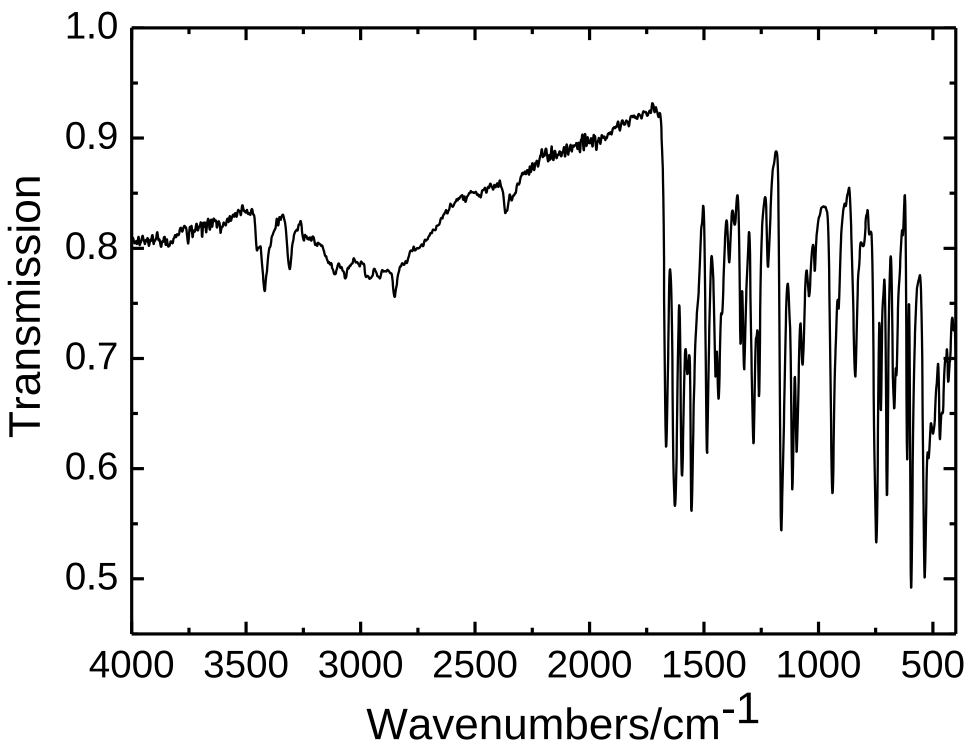

Figure S21: ATR-IR spectrum of **4**
